# An Integrated and Robust Vision System for Internal and External Thread Defect Detection with Adversarial Defense

**DOI:** 10.3390/s25185664

**Published:** 2025-09-11

**Authors:** Liu Fu, Leqi Li, Gengpei Zhang, Zhihao Jiang

**Affiliations:** The School of Electronic Information and Electrical Engineering, Yangtze University, East Campus, Jingzhou 434100, China; fliu68596@gmail.com (L.F.); lileqi2023@outlook.com (L.L.); judgebill@126.com (G.Z.)

**Keywords:** thread defect detection, lightweight neural network, alpha channel attack, YOLO-based optimization, image data augmentation

## Abstract

In industrial automation, detecting defects in threaded components is challenging due to their complex geometry and the concealment of micro-flaws. This paper presents an integrated vision system capable of inspecting both internal and external threads with high robustness. A unified imaging platform ensures synchronized capture of thread surfaces, while advanced image enhancement techniques improve clarity under motion blur and low-light conditions. To overcome limited defect samples, we introduce a generative data augmentation strategy that diversifies training data. For detection, a lightweight and optimized deep learning model achieves higher precision and efficiency compared with existing YOLO variants. Moreover, we design a dual-defense mechanism that effectively mitigates stealthy adversarial perturbations, such as alpha channel attacks, preserving system reliability. Experimental results demonstrate that the proposed framework delivers accurate, secure, and efficient thread defect detection, offering a practical pathway toward reliable industrial vision systems.

## 1. Introduction

Threaded components are fundamental units widely used in modern industrial equipment for connection and power transmission, playing crucial roles across key sectors such as machinery, automotive, aerospace, energy, and rail transportation. In various assembly structures, threads are not only responsible for mechanical connection and sealing alignment but are also directly related to the overall structural reliability and safety. Due to their complex geometric structure and stringent machining precision requirements, even minute defects—such as broken threads, burrs, cracks, and corrosion—can lead to loosening, sealing failure, or even catastrophic mechanical breakdowns. Therefore, effective defect detection for industrial threaded parts is vital for ensuring product quality, improving assembly consistency, and reducing equipment failure rates.

Beyond the most primitive visual inspection, traditional defect detection methods for threads largely rely on contact-based measurements, such as thread gauges (e.g., plug and ring gauges), calipers, micrometers, and profilometers with mechanical probes [1,2]. These techniques involve manual or semi-automatic operations to identify issues like dimensional deviations or machining defects. However, they suffer from low efficiency, reliance on operator experience, and difficulty detecting micro or structural defects—especially in reflective surfaces, deep cavities, or mass production settings—making them inadequate for modern manufacturing demands of high precision, efficiency, and automation.

In recent years, non-contact inspection technologies for industrial components have seen substantial advancements, becoming mainstream due to their non-destructive, automated, and high-accuracy advantages. These include computer vision inspection [3,4,5], laser scanning [6,7,8], X-ray [9,10], and optical techniques [11,12,13]. Among them, computer vision-based defect detection stands out for its lightweight nature, easy deployment, high accuracy, and low cost, making it the most general and extensible solution within non-contact inspection methods.

Image processing is the foundational technology in computer vision and has been widely adopted across visual inspection tasks. Notably, dynamic image deblurring has made significant progress through deep learning. Jang et al. (2025) [14] proposed DSANet, a deep supervision attention network leveraging a ConvLSTM encoder-decoder and frequency-domain constraints for precise recovery of blurred regions. Ren et al. (2022) [15] introduced a spatially variant neural network for dynamic scene blur, combining CNN and RNN to better model complex blur patterns. Gao et al. (2019) [16] presented a parameter-sharing network with nested skip connections and released a benchmark dataset for dynamic deblurring. Zhang et al. (2023) [17] adopted a flow-guided multi-scale RNN to improve fine detail recovery, while Chen et al. (2023) [18] proposed a CNN–Transformer hybrid model using stripe attention and cross-layer feature fusion, achieving state-of-the-art performance on multiple benchmarks.

Illumination normalization, a critical step in image preprocessing, enhances robustness under suboptimal lighting for tasks such as enhancement, detection, and recognition. Vasluianu et al. (2024) [19] introduced Ambient Lighting Normalization (ALN) and a corresponding dataset Ambient6K using frequency-domain fusion to restore shadowed regions. Dias Da Cruz et al. (2020) [20] proposed a learning framework with partially unachievable autoencoder objectives for better illumination-invariant representation. Huang et al. (2023) [21] proposed Transition-Constant Normalization (TCN) for stable enhancement under varying exposures. Rad et al. (2020) [22] developed Adaptive Local Contrast Normalization (ALCN), dynamically predicting normalization parameters to boost recognition in complex lighting. Goswami (2020) [23] designed a deployable deep method for correcting uneven lighting in RGB images, showing good generalization in real-world scenarios.

With the advancement of deep learning, many vision-based defect detection systems have integrated deep neural networks to improve recognition accuracy and robustness in complex settings. Jiang et al. (2024) [24] combined GAN and YOLO for generating and detecting internal thread defects, achieving 94.27% and 93.92% accuracy for internal and external threads, respectively. Dou et al. (2024) [25] developed a multi-camera inspection system incorporating lighting optimization and cylindrical image stitching, enabling efficient and visual thread defect localization. Xu et al. (2023) [26] proposed an enhanced YOLOv5 for bearing defect detection using C2f, SPD modules, and CARAFE upsampling, achieving 97.3% mAP and 100 FPS. Wu et al. (2025) [27] introduced RBS-YOLO, a lightweight version of YOLOv5 for casting defects, balancing accuracy and complexity. Patil (2024) [28] compared YOLOv5 and YOLOv8 for nut thread presence detection, highlighting YOLOv8′s superior speed and accuracy. Lang et al. (2022) [29] proposed MR-YOLO by integrating MobileNetV3, SE attention, and Mosaic augmentation, improving efficiency and accuracy. Tabernik et al. (2019) [30] designed a DNN-based segmentation model with few-shot learning support. Wang et al. (2022) [31] introduced Defect Transformer (DefT), a hybrid CNN–Transformer model that captures both local details and global semantics, enhancing detection robustness.

In the broader field of adversarial machine learning, numerous studies have revealed the vulnerability of deep neural networks. Classical gradient-based methods, such as FGSM and PGD [32,33,34], can mislead classifiers by applying subtle perturbations at the pixel level. Patch attacks [35,36,37] introduce locally high-contrast patterns into images, digitally or physically occluding critical regions, while color channel perturbation methods [38,39] create global distortions by altering the ratios of RGB channels. These attacks have been shown to cause significant drops in detection and classification accuracy, highlighting the lack of robustness in current visual recognition systems. However, most existing research has either focused on generic computer vision benchmark datasets or explored only limited types of perturbations, with little attention paid to the specific requirements of industrial visual inspection. In particular, very few studies have investigated highly covert and practically deployable threats—alpha channel attacks [40,41]. As a representative form of implicit perturbation, alpha channel attacks can inject imperceptible interference signals through the transparency channel of an image, substantially degrading model perception without altering the image as observed by the human eye. Since most industrial detection models by default process RGBA inputs without stripping the transparency channel, this type of attack is both easy to deploy and difficult to detect, making it a critical security threat to industrial vision systems. In high-security applications such as automatic thread inspection and screening, any form of image perturbation that is not promptly identified may lead to downstream assembly precision errors or failures in quality control. Therefore, developing detection frameworks with adversarial defense capabilities is not only essential for enhancing system robustness but also constitutes a fundamental security infrastructure in the advancement of industrial intelligence.

This study extends prior work in small-object detection, multi-scale adaptability, and edge-device deployment while introducing a security-aware perspective to strengthen real-world robustness. The main contributions are as follows:

1. A dual-mode industrial image acquisition setup is constructed for internal and external threads, integrating fisheye lenses and HD cameras to solve structural complexity and switching inefficiencies in traditional systems.
2. MLWNet and DarkIR are employed for dynamic deblurring and illumination normalization, ensuring high-quality inputs. A residual diffusion denoising model (RDDM) is introduced for generating and augmenting thread defect samples.
3. A novel detection model, SLF-YOLO, is developed by integrating SC_C2f, Light-SSF_Neck, and FIMetal-IoU loss, outperforming YOLOv5s to YOLOv10s while remaining suitable for real-time edge deployment.
4. A defense mechanism, the Histogram–MSE Defense Model (HMDM), is proposed to counter alpha channel attacks. By combining histogram overlap analysis with MSE-based detection, HMDM effectively identifies and mitigates input-level adversarial perturbations, enhancing the robustness and security of the system.

The remainder of this paper is organized as follows. Section 2 presents the overall principle and architecture of the proposed integrated vision system, including image acquisition, preprocessing, data augmentation, defect detection, and adversarial defense mechanisms. Section 3 describes the experimental setup and evaluates the performance of the system in terms of image enhancement, defect detection accuracy, robustness against adversarial perturbations, and comparative analysis with baseline models. Section 4 discusses the contributions of individual modules, analyzes ablation studies, and further investigates the internal mechanisms and practical implications of adversarial perturbations. Finally, Section 5 concludes the paper by summarizing the main findings and outlining potential directions for future research.

## 2. Principle and System Overview

**Figure 1:**
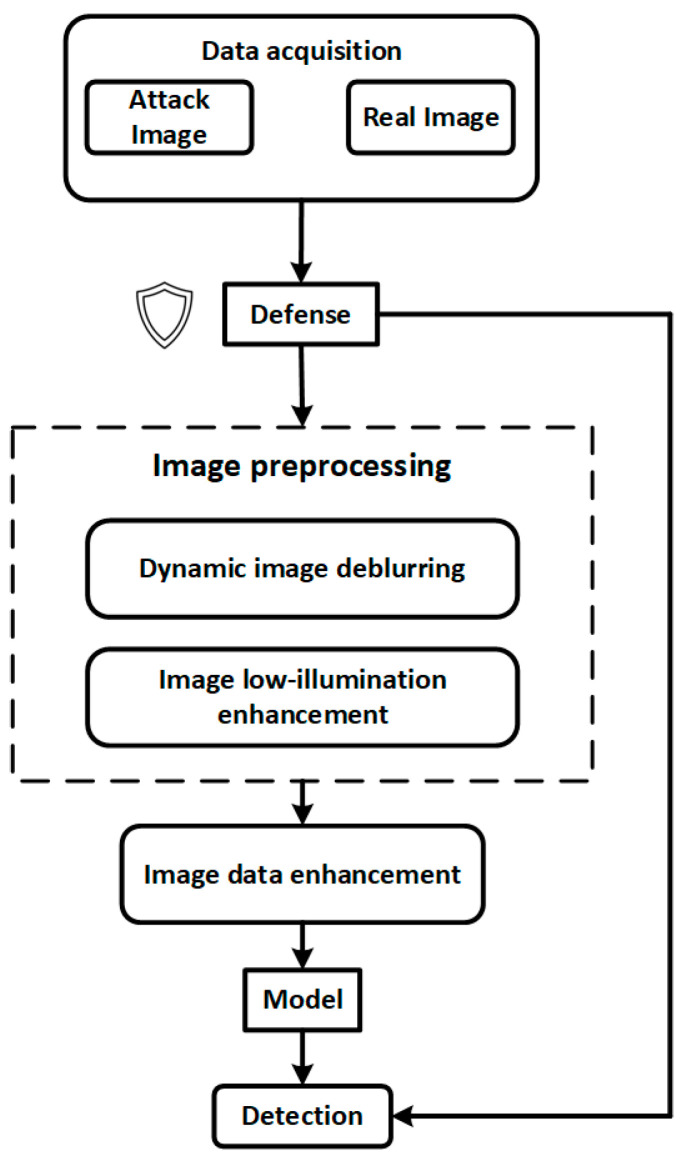
Overall system architecture.

Figure 1 illustrates the overall processing workflow of the proposed internal and external thread defect detection system. From the initial data acquisition stage, the system integrates multi-level image enhancement and security defense mechanisms to improve model robustness and detection stability under complex industrial interference conditions.

The system begins by acquiring multi-source input data from industrial environments, which includes both normal images and potential adversarial samples. To counteract threats such as alpha channel attacks, transparent padding interference, and adversarial patches, the input data is first processed by a lightweight security defense module. This module performs perturbation suppression, anomaly filtering, and input resizing to preliminarily eliminate explicit attack characteristics and prevent malicious samples from entering the core model pipeline.

Next, the data flows into the image preprocessing submodule, which consists of two processing paths:

(1) Dynamic deblurring, designed to mitigate motion blur caused by device vibration or camera instability, thereby enhancing the visibility of thread edges and defect boundaries;
(2) Low-light enhancement, targeted at restoring image quality in dark cavities such as internal threads, utilizing brightness normalization and edge detail enhancement to improve model perception.

The preprocessed images are then passed to the Image Data Enhancement module, where a residual diffusion denoising model (RDDM) is employed for defect diversity modeling and synthetic data generation. This enhances the model’s generalization capability and defect coverage under limited-sample conditions.

The enhanced image data is subsequently fed into a deep object detection network for defect identification and localization. The detection results are also fed back into a front-end security monitoring module, enabling output-based anomaly detection. For instance, abrupt changes in the number of bounding boxes or unusual clustering of defect categories can trigger an alarm or pause the model response, thus forming a closed-loop industrial vision security chain with perception, diagnosis, and response capabilities.

Overall, the proposed workflow not only ensures high detection accuracy but also integrates a three-stage defense pipeline—pre-processing, mid-processing, and post-processing—offering comprehensive protection against adversarial perturbations, transparent padding, and real-world industrial interference. This design ensures strong industrial adaptability and controllable system security.

### 2.1. Image Acquisition

As the first and foundational stage in the internal and external thread defect detection pipeline, the quality, viewpoint completeness, and spatial accuracy of thread image acquisition directly determine the upper performance limits of subsequent feature extraction and object recognition algorithms. To obtain high-fidelity, full-coverage, and unobstructed image inputs, this study designs a unified image acquisition system tailored to industrial field applications, capable of handling both internal and external threads.

In conventional industrial inspection systems, internal and external threads are typically imaged using separate devices and workflows due to their distinct structural positions: external threads are usually captured via multi-camera setups arranged around the object, while internal threads require endoscopic probes to access deep cavities. These differences in installation, illumination strategies, and imaging paths result in complex hardware configurations, high switching costs, and low efficiency in batch inspections.

**Figure 2:**
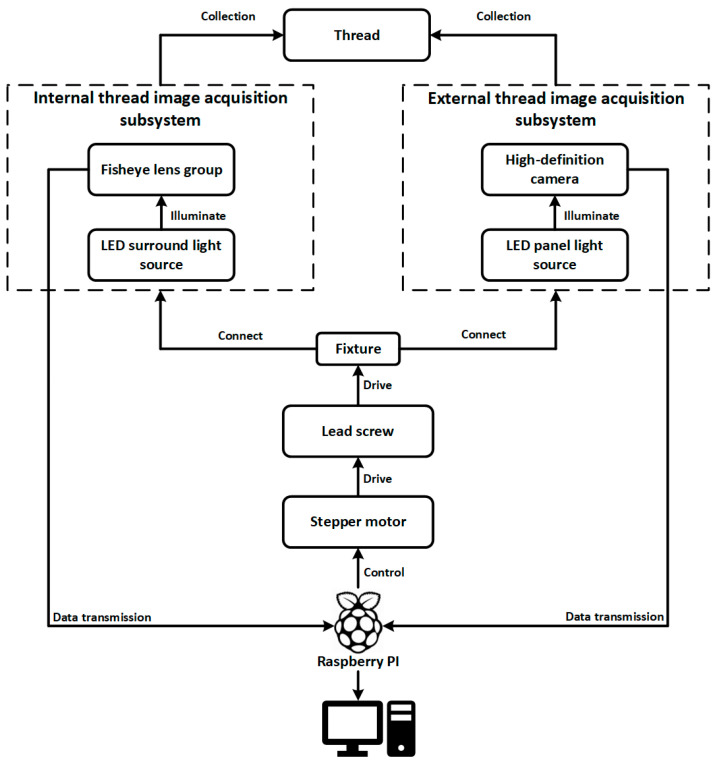
System architecture of the industrial internal and external thread image acquisition platform.

To address these challenges, we propose an integrated image acquisition architecture for industrial thread defect detection, as illustrated in Figure 2. The system is constructed around the multi-angle structural characteristics of threaded components, incorporating independent subsystems for internal and external thread image capture. These subsystems are synchronized using stepper motors, transmission mechanisms, and an embedded control platform to achieve precise, coordinated acquisition of dynamic thread targets.

The workpiece is fixed on a central fixture driven by a lead screw mechanism powered by a stepper motor, enabling linear axial movement. The displacement of the motor and the image acquisition signals are orchestrated by a Raspberry Pi-based control unit, ensuring closed-loop synchronization of image triggering, motion control, and data transmission.

For internal thread imaging, the system employs a fisheye lens group combined with an LED ring light source, enabling wide-angle imaging and uniform circumferential illumination within the cavity. This setup effectively mitigates the challenges of light-shadow blind spots and angle occlusion along the thread’s inner wall. For external thread imaging, a high-definition industrial camera coupled with an LED panel light source is used to achieve full circumferential coverage of the outer surface with parallel illumination, suitable for rod-like components such as screws and spindles.

**Figure 3:**
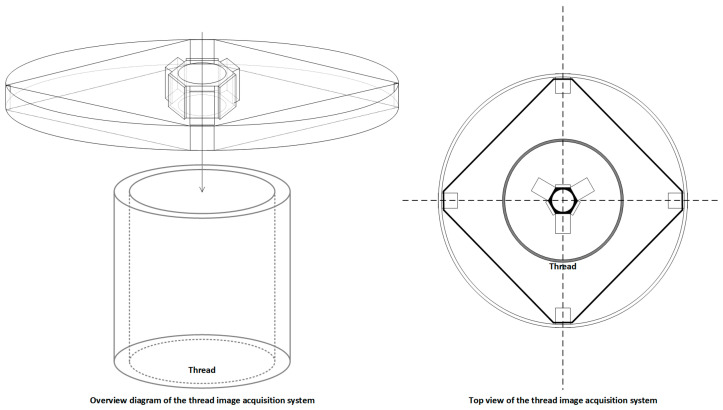
Internal and external thread imaging hardware design.

Both subsystems are connected to the main control platform via the fixture linkage structure. The acquired images are transmitted in real time through the Raspberry Pi to a backend detection host, where the defect recognition network operates. Thanks to this dual-subsystem collaborative design, the platform supports unified, adjustable, and multi-angle thread image capture, forming a stable data foundation for high-precision vision-based defect detection. The hardware structure is depicted in Figure 3.

**Figure 4:**
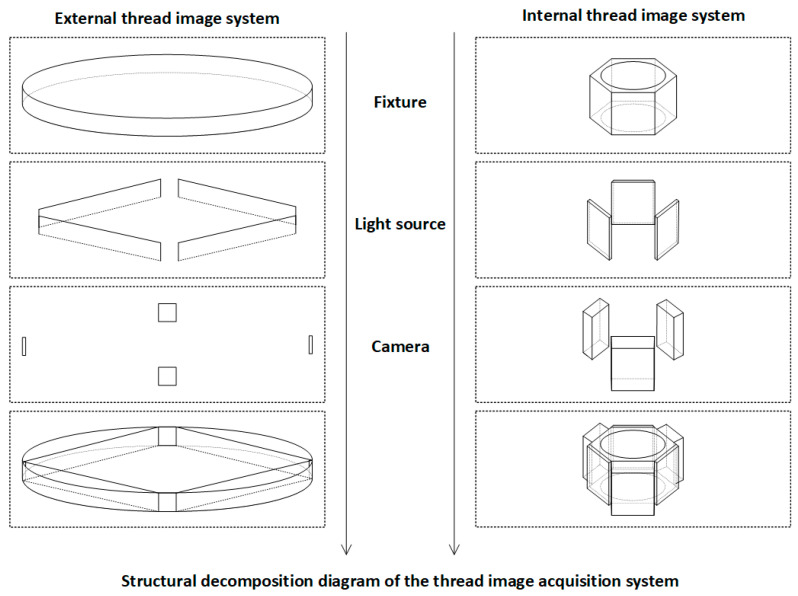
System structural breakdown and design details.

The combination of dual imaging subsystems with a central lead screw lifting platform and multi-angle mounting modules allows for the simultaneous and integrated acquisition of both internal and external wall images on a single device. This approach not only improves assembly consistency and reduces platform complexity but also ensures spatial-temporal alignment and structural consistency of the images. As a result, the system provides standardized input sources for downstream defect detection models, significantly enhancing overall detection accuracy, system stability, and industrial deployability. Detailed component illustrations are shown in Figure 4.

### 2.2. Dynamic Deblurring

In industrial visual inspection scenarios, image blur is a prevalent issue—particularly in regions with complex geometries such as metallic threads and tubular cavities. This type of degradation often arises from equipment vibration, insufficient exposure, or motion-induced defocus, leading to pronounced directional motion blur. Such blur is typically accompanied by attenuation of high-frequency textures, edge smearing, and structural distortion, which severely compromises image clarity and limits its usability in defect detection, depth estimation, and 3D modeling tasks. To address this, a dynamic deblurring module is introduced at the image preprocessing stage, forming a comprehensive image enhancement pipeline in conjunction with the illumination normalization module.

We adopt MLWNet (Multi-scale Network with Learnable Wavelet Transform) [42] as the backbone of the dynamic deblurring module. MLWNet incorporates learnable two-dimensional discrete wavelet transform (2D-LDWT) and a multi-scale semantic fusion mechanism to effectively capture blur features across different scales and orientations. Unlike conventional spatial-domain networks, MLWNet introduces frequency modeling during the feature extraction stage, with a specific focus on restoring high-frequency details and edge structures that are severely degraded by motion blur.

At the modeling level, wavelet transform decomposes an image $f\left(t\right)$ into a low-frequency approximation term and multiple high-frequency directional components:

(1) $$f\left(t\right)=\sum_{j>j_{0}}\sum_{k}d_{j,k}\psi_{j,k}\left(t\right)+\sum_{k}c_{j_{0},k}\phi_{j_{0},k}\left(t\right)$$

where $d_{j,k}$ represents the high-frequency detail coefficients, and $c_{j_{0},k}$ denotes the low-frequency approximation coefficients. This decomposition provides multi-scale frequency resolution capabilities.

In the network, high-pass and low-pass filters ${\;\vec{a}}_{1}$ and ${\;\vec{a}}_{0}$ are used to perform recursive convolution operations, resulting in four sets of 2D filtered wavelet components—LL, LH, HL, and HH—which are concatenated to form a four-channel wavelet convolution kernel $K_{w}$.

To ensure reversibility and energy conservation during both the forward and inverse wavelet transformations, Perfect Reconstruction Constraints are introduced:

(2) $$A_{0}\left(-z\right)S_{0}\left(z\right)+A_{1}\left(-z\right)S_{1}\left(z\right)=0,{\;A}_{0}\left(z\right)S_{0}\left(z\right)+A_{1}\left(z\right)S_{1}\left(z\right)=2$$

In terms of architecture, the input image is first processed through multiple Simple Encoder Blocks (SEBs) to extract shallow features and perform multi-scale downsampling. The central module, Wavelet Fusion Block (WFB), employs Learnable Wavelet Nodes (LWNs) to conduct forward wavelet transformation and directional detail modeling. Frequency-domain features are extracted using depthwise separable convolutions and channel reconstruction and are then fused back into the spatial domain through residual connections. The decoding phase uses several Wavelet Head Blocks (WHBs) for progressive feature upsampling and image clarity restoration, ultimately producing a high-resolution deblurred image.

For the training strategy, the network is optimized using two types of loss functions: the multi-scale loss $L_{\mathrm{multi}}$, which supervises the pixel-wise discrepancies between outputs at different scales and the ground truth (GT), and the wavelet reconstruction loss $L_{\mathrm{wavelet}}$, which ensures consistent frequency-domain modeling by the Learnable Wavelet Node (LWN) module. The final total loss is defined as:

(3) $$L_{\mathrm{total}}=L_{\mathrm{multi}}+\lambda \cdot L_{\mathrm{wavelet}}$$

### 2.3. Illumination Normalization

In real-world industrial inspection environments, the surfaces of threaded metallic components frequently exhibit strong shadows, specular highlights, and non-uniform exposure due to the periodic geometry, high reflectivity of materials, and significant variations in ambient lighting. These conditions impose substantial challenges to vision-based defect detection models, often leading to unstable or inaccurate predictions.

To enhance the consistency of input images and improve illumination robustness, this study introduces an image-level illumination normalization module during the data preprocessing stage.

This study adopts an illumination processing framework based on the Retinex theory [43], which models the observed image $I\left(x,y\right)$ as the product of a reflectance component $R\left(x,y\right)$ and an illumination component $L\left(x,y\right)$:

(4) $$I\left(x,y\right)=R\left(x,y\right)\cdot L\left(x,y\right)$$

In the logarithmic domain, this multiplicative relationship is transformed into an additive model for easier processing:

(5) $$\log I\left(x,y\right)=\log R\left(x,y\right)+\log L\left(x,y\right)$$

To extract reflectance components that are more sensitive to subtle structural defects, we propose a local brightness-constrained enhancement strategy, which combines local contrast amplification with gamma compression into a unified normalization framework. This approach performs spatial mean filtering in the brightness channel to suppress low-frequency illumination artifacts while adaptively adjusting the contrast range of the image. It enhances the visibility of fine textures and edge-related features that are critical for defect detection.

For practical implementation, we adopt the fast and deployable Retinex by Adaptive Filtering (RAF) method as the primary algorithm, combined with gamma compression:

(6) $$I_{\mathrm{norm}}\left(x,y\right)={\left(\frac{I\left(x,y\right)}{G_{\sigma }\left(I\right)}\right)}^{\gamma }$$

Here, $G_{\sigma }\left(I\right)$ denotes the Gaussian-smoothed output of the image brightness channel, and γ controls the non-linear compression of brightness. This method suppresses overexposure in locally bright areas and enhances contrast in low-illumination or occluded regions. It is particularly effective for inner surfaces of metallic threads, where reflective lighting often causes pseudo-defect patterns due to structural highlights.

**Figure 5:**
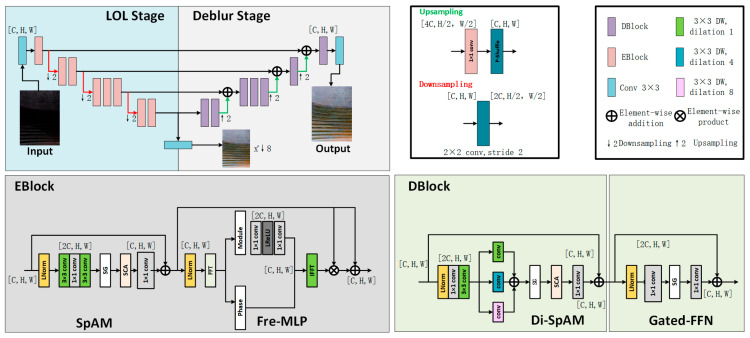
Architecture of the proposed dual-stage DarkIR network for low-light enhancement and image deblurring.

The network architecture, as illustrated in Figure 5, adopts a dual-stage cooperative design aimed at simultaneously addressing low-light enhancement and image deblurring. The overall network consists of two distinct stages: the Low-Light Enhancement Stage (LOL Stage) and the Deblurring Stage (Deblur Stage). Both stages utilize a symmetric encoder–decoder architecture with multi-scale feature extraction capabilities and are connected via skip connections to ensure efficient transmission and fusion of feature information.

The LOL Stage focuses on restoring brightness and enhancing fundamental details in low-illumination input images, thereby improving overall visibility. Building on the enhanced outputs, the Deblur Stage further strengthens edge structures and restores texture details, effectively compensating for blur caused by low lighting or acquisition jitter.

In terms of module design, DarkIR integrates an EBBlock (Enhancement Block) into the LOL Stage. This block contains two key submodules:

(1) The SpAM (Spatial Attention Module) enhances local responses via spatial attention mechanisms, improving brightness expression under uneven lighting conditions;
(2) The Fre-MLP (Frequency-aware MLP) module centers on frequency-domain modeling, leveraging frequency information to preserve fine details and reduce noise—especially suited for handling high-frequency regions such as industrial surface textures.

The EBBlock output is fused with the main feature stream via residual connections, ensuring stability throughout the enhancement process.

In the Deblur Stage, DarkIR incorporates the DBlock module for high-quality restoration of blurred regions. DBlock consists of:

(1) Di-SpAM (Dilated Spatial Attention Module), which uses dilated convolutions to enlarge the receptive field and capture edge cues in low-contrast backgrounds;
(2) Gated-FFN (Gated Feed-Forward Network), which enables discriminative modeling between blurred and sharp regions during information propagation, thus better preserving structural integrity and suppressing artifacts.

The entire network employs standard strided convolutions and transposed convolutions for downsampling and upsampling, respectively. Additionally, skip connections between multiple scales enable the flow of semantic and fine-grained visual information across layers, further enhancing the network’s multi-scale perceptual capability.

### 2.4. Image Data Augmentation

In industrial internal and external thread defect detection tasks, the acquisition of high-quality and representative image samples is often constrained by factors such as complex spatial structures, reflective metallic surfaces, and occluded viewpoints. These limitations result in a scarcity of annotated data, which restricts the robustness and generalization capability of deep learning-based detection models.

To address the limited-data problem, this study introduces a high-fidelity defect image generation framework based on diffusion modeling during the data augmentation phase. Specifically, a residual diffusion denoising model (RDDM) is employed to perform conditional sampling on original thread images, thereby simulating a broader distribution of diverse and representative defect types.

The RDDM method follows the classical forward–reverse diffusion modeling paradigm. In the forward process, the original image $x_{0}$ is progressively injected with Gaussian noise via a Markov chain, expressed as:

(7) $$q\left(x_{t}|x_{t-1}\right)=N\left(x_{t};\sqrt{1-\beta_{t}}\cdot x_{t-1},\beta_{t}\cdot I\right)$$

where $\beta_{t}$ is the diffusion coefficient at time step t, controlling the noise injection intensity. The reverse generation process is guided by a residual-conditioned denoising predictor to iteratively reconstruct the original signal, defined by the target distribution:

(8) $$p_{\theta }\left(x_{t-1}|x_{t},c\right)=N\left(x_{t-1};\mu_{\theta }\left(x_{t},c,t\right),\sum_{\theta }\left(x_{t},c,t\right)\right)$$

Here, c denotes the defect category label, and $\mu_{\theta }$ and $\sum_{\theta }$ are the conditional mean and variance estimated by the learned model.

Unlike traditional DDPM approaches, RDDM introduces a residual prediction strategy, which does not directly predict the original image $x_{0}$ but instead predicts the residual information:

(9) $${\hat{r}}_{\theta }\left(x_{t},c,t\right)=x_{t}-\mu_{\theta }\left(x_{t},c,t\right)$$

This residual-based formulation effectively mitigates issues such as edge blurring and texture degradation and is particularly suitable for enhancing fine-grained defects like thread breaks, burrs, and contamination in industrial images.

In this study, we utilize real-world internal and external thread defect samples as priors. The RDDM is conditioned on defect labels to perform diffusion-based sampling, generating high-fidelity defect images that not only retain geometric consistency with real samples but can also simulate diverse defect types across different sampling iterations.

### 2.5. Defect Detection

**Figure 6:**
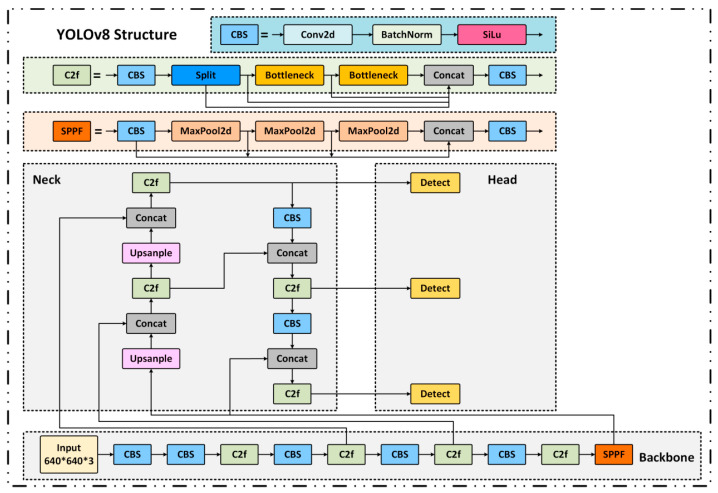
Architecture of the baseline YOLOv8 detection network. The structure is redrawn with reference to “What is YOLOv8: An In-Depth Exploration of the Internal Features of the Next-Generation Object Detector” [47].

In this study, the YOLO (You Only Look Once) [44,45] series is adopted as the foundational framework for metallic surface defect detection due to its high inference efficiency as a single-stage object detector. Among them, YOLOv8 [46] significantly enhances feature extraction and multi-scale fusion capabilities by introducing the C2f module and BiFPN structure. However, challenges remain in accurately detecting small-scale defects under complex industrial backgrounds. The baseline network architecture is illustrated in Figure 6.

To overcome these limitations, we propose a lightweight enhanced architecture named SLF-YOLO, which integrates three key components:

(1) The SC_C2f module for improved channel-wise feature fusion;
(2) The Light-SSF_Neck structure for efficient multi-scale aggregation;
(3) A novel loss function termed FIMetal-IoU, designed to optimize bounding box regression under industrial constraints.

**Figure 7:**
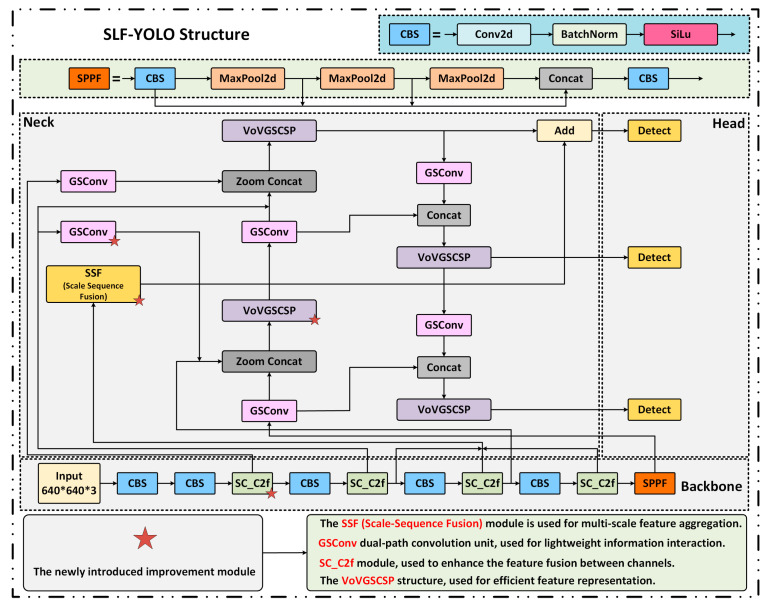
Architecture of the proposed SLF-YOLO detection network with modular enhancements.

The overall structure of SLF-YOLO is shown in Figure 7. In the backbone, the SC_C2f module incorporates a Star Block for enhanced feature interaction and leverages a Channel-Gated Linear Unit (CGLU) activation to enable fine-grained dynamic channel selection.

The key computation formulas are defined as follows:

To optimize information flow and multi-scale feature fusion in the Neck stage, we adopt the Light-SSF_Neck structure. Its core component, the GSConv module, fuses Standard Convolution (SC) and Depthwise Separable Convolution (DSC) to enhance channel-wise information exchange via dual-path computation:

(10) $$Y_{\mathrm{GSConv}}=\mathrm{Shuffle}\left(\mathrm{Concat}\left(Y_{\mathrm{SC}},Y_{\mathrm{DSC}}\right)\right)$$

Additionally, the Scale-Sequence Fusion (SSF) module extracts multi-scale features from P3, P4, and P5 and applies convolution, upsampling, and 3D convolutional fusion to construct cross-scale contextual representations:

(11) $$Y_{\mathrm{fusion}}=\mathrm{Conv}3D\left(\mathrm{Concat}\left(Y_{P3},Y_{P4\to P3},Y_{P5\to P4}\right)\right)$$

To further enhance localization accuracy, we propose a novel loss function called FIMetal-IoU, which introduces an auxiliary box mechanism and piecewise weighting scheme into the IoU computation. The auxiliary box IoU is defined as:

(12) $${\mathrm{IoU}}_{\mathrm{Inner}}=\frac{{\mathrm{inter}}_{\mathrm{inner}}}{{\mathrm{union}}_{\mathrm{inner}}}$$

Based on this, we apply piecewise weighting to different IoU intervals, and the final loss function is expressed as:

(13) $$\left|\mathrm{FIMetal}-\mathrm{IoU}\right|=\left\{\begin{array}{cc} 0, & {\mathrm{IoU}}_{\mathrm{Inner}}<d\\ \frac{{\mathrm{inter}}_{\mathrm{inner}}-{\mathrm{union}}_{\mathrm{inner}}\cdot d}{{\mathrm{union}}_{\mathrm{inner}}}\left(u-d\right), & d<{\mathrm{IoU}}_{\mathrm{Inner}}<u\\ 1, & {\mathrm{IoU}}_{\mathrm{Inner}}>u \end{array}\right.$$

### 2.6. Adversarial Attacks on Image-Based Systems

Adversarial attacks on images represent a major security threat to deep learning-based vision systems. Their core objective is to induce incorrect predictions or outputs by introducing subtle but intentionally crafted perturbations to the input image, thereby compromising the system’s robustness and trustworthiness.

Based on their implementation methods and attack effects, adversarial attacks can be categorized into various types. Among them, alpha attacks, CCP (color channel perturbation), and patch attacks are three representative methods, as summarized in Table 1.

Alpha attacks combine high imperceptibility with extremely strong attack capability, making them one of the most severe threats to current image recognition systems. Therefore, it is essential to develop high-sensitivity defense mechanisms specifically targeting this form of attack.

Although CCP and Patch attacks pose relatively lower threats, they still introduce practical security risks—especially in large-scale deployments of vision systems, where adversaries may exploit their low complexity to achieve rapid system compromise.

Accordingly, the design of image-level security defense strategies should be based on a multi-layered security framework incorporating

(1) A robust model architecture design;
(2) Adversarial training techniques;
(3) Multimodal detection methods.

These measures collectively enhance the model’s adversarial robustness and resistance to diverse threat vectors in real-world industrial environments.

An alpha channel attack is a stealthy adversarial method based on the transparency dimension of image representation. In recent years, it has emerged as a highly concealed and engineering-feasible input-level threat in security-sensitive industrial visual inspection systems. This method exploits the structural vulnerabilities in image processing pipelines by embedding adversarial perturbations into the alpha (transparency) channel of standard image formats (e.g., PNG), which are typically not perceived by human vision systems.

While alpha channel manipulations are generally unsupported in human-viewing libraries, they remain invisible yet processable in industrial vision pipelines that rely on image pre-processing frameworks such as OpenCV, TensorRT, PIL, or PyTorch. As a result, these perturbations bypass typical input validation and are treated as valid tensors by deep neural networks, allowing attackers to create stealthy adversarial samples without altering pixel color or brightness. The attack is formulated as:

(14) $$x_{\mathrm{adv}}=\left(1-\alpha \right)⊙x_{\mathrm{orig}}+\alpha ⊙\delta$$

Here, $x_{\mathrm{orig}}$ is the original industrial image, δ is the adversarial perturbation map, and α is the transparency mask controlling the blend intensity. This operation introduces controllable perturbations through the alpha channel without altering the color and brightness distribution of the image pixels. It effectively interferes with the responses of the model in the convolution feature extraction of the previous layer, especially having a significant interference effect on the periodic textures, gap edges, and multi-scale concave structures in the threaded images.

To ensure imperceptibility and maintain image quality, the perturbation design is subject to the following constrained optimization:

(15) $$\underset{\delta }{\min}L\left(f\left(x_{\mathrm{adv}}\right),y_{\mathrm{target}}\right)+\lambda \cdot \|\delta {\|}_{p}$$

Here, $f\left(\cdot \right)$ denotes the target detection model, and $y_{\mathrm{target}}$ is the desired misclassification output. The loss function is used to guide the model’s output to deviate from the original detection result, while the regularization term controls the magnitude of the perturbation to meet the perceptual constraints. This form enables attackers to deceive the model under multiple task settings, including common industrial errors such as misclassification of defect types, deviation in position regression, and decreases in the confidence level of bounding boxes. $L\left(\cdot \right)$ is the loss function guiding the attack, and $\|\delta {\|}_{p}$ is the perturbation norm, regularized by λ.

To preserve perceptual quality and system integrity, the following constraints are enforced:

(16) $$\|\delta {\|}_{\infty }<\epsilon ,\;\alpha <\tau$$

where ϵ is the maximum perturbation bound and τ is the upper bound for transparency. Both are typically set below 0.1 to avoid triggering quality-based preprocessing thresholds and to ensure compatibility with image format standards.

In threaded defect detection applications, alpha channel perturbations have been experimentally demonstrated to cause typical false detections and missed detections at the output of neural network models. Specifically, such perturbations can lead to positional drift in defect localization (e.g., misaligned gap detection), destruction of edge integrity, and interference from structurally repetitive regions.

Notably, even under static image dimensions, the adversarial effect exhibits strong transferability across samples and models, indicating cross-model attack capability. Given that most industrial lightweight detection networks—such as the SLF-YOLO model proposed in this study—do not explicitly regulate or suppress four-channel inputs, alpha-based perturbations present a realistic deployment risk, warranting serious attention during the system design phase for security reinforcement.

In conclusion, alpha channel attacks represent a form of implicit input-level perturbation characterized by high stealthiness, cross-model adaptability, and engineering feasibility.

They have become an emerging but critical security threat in industrial-grade object detection systems. This work constructs an attack modeling framework tailored to thread-structured images and systematically uncovers the disruptive mechanisms and misleading effects of alpha perturbations on convolutional feature responses.

Furthermore, this study highlights the necessity of removal or masking mechanisms for the alpha channel in the image preprocessing pipeline. Combined with the proposed Histogram–MSE Defense Model (HMDM), the approach provides a practical reference for alpha channel risk assessment and mitigation strategies during the pre-deployment stage of industrial vision systems, aiming to (i) reduce vulnerability to adversarial inputs at the source and (ii) enhance the overall robustness and security of the system.

While the proposed Histogram–MSE Defense Model (HMDM) effectively mitigates alpha channel attacks by detecting discrepancies in the image histogram and pixel-level MSE, its statistical framework is inherently adaptable to other types of adversarial perturbations. For example, high-frequency noise attacks, often targeted at disrupting fine-grained textures, could be detected using frequency-domain features such as the Fourier Transform. Similarly, patch attacks, which aim to locally occlude regions of the image, could be detected by analyzing local discrepancies in image regions through local MSE or SSIM metrics. Furthermore, color channel perturbations could be identified by detecting abnormal shifts in the image’s color histogram or in the LAB color space.

## 3. Experiments

### 3.1. Dynamic Deblurring

In industrial thread defect detection tasks, image blur is one of the key interference factors that significantly affects the accuracy of detection models. This issue is particularly pronounced in external threads, which often exhibit periodic structural features and small-scale defects. Even minor motion blur can severely degrade edge sharpness and target contrast, resulting in localization deviation, reduced confidence scores, or even complete miss detections.

**Figure 8:**
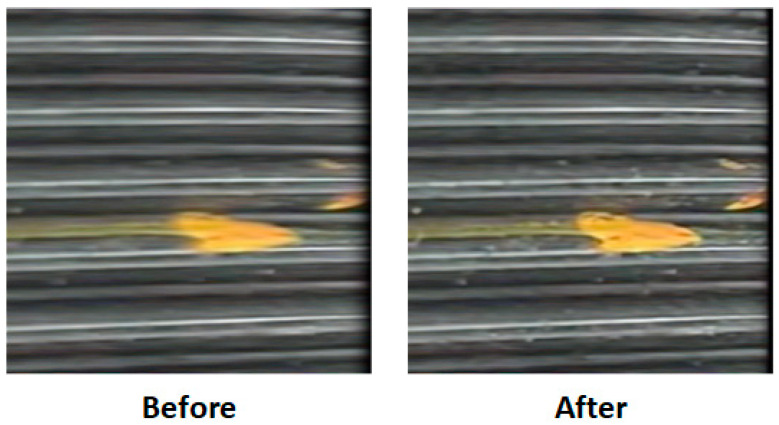
Comparison of thread corrosion defect images before and after dynamic deblurring.

Figure 8 illustrates a visual comparison of typical external thread corrosion defect images before and after dynamic deblurring.

The left image represents the original unprocessed input, where the high-speed rotation of the threaded pipe or minor camera vibration during image acquisition has introduced noticeable motion blur. This is evident in the blurred boundary of the corrosion spot (yellow area) and the streaking of background thread lines.

In contrast, the right image, processed using the proposed dynamic modeling-based deblurring enhancement method, shows sharpened defect edges, restored periodic thread patterns, and significantly improved contrast and texture clarity.

The proposed dynamic deblurring module integrates residual attention-based local blur recognition with a frequency-domain compensation mechanism, allowing for precise localization of degraded regions and adaptive restoration. While maintaining global structural consistency, it significantly enhances the separability and visual saliency of corrosion boundaries.

This method also demonstrates strong generalizability to common local blur issues in industrial thread imagery, such as exposure trailing caused by illumination variation and misalignment between motion speed and sampling rate.

It thus provides a robust and adaptable solution to blur-related challenges in real-world industrial inspection scenarios.

### 3.2. Impact of Illumination Normalization on Detection Accuracy

We conducted a visual comparison on internal thread images commonly found in industrial scenarios before and after enhancement.

In the left image (originally captured under extreme low-light conditions), the thread structure is nearly completely obscured by shadows, exhibiting severe black suppression and significant illumination non-uniformity.

The right image, processed using DarkIR, shows substantial improvements in detail visibility, with the hierarchical thread structures clearly recovered and the overall dynamic brightness range significantly expanded.

As an end-to-end deep learning method tailored for ultra-low-light image restoration, DarkIR adopts a learnable nonlinear mapping structure that enhances brightness while suppressing color distortion and noise amplification, issues that are frequently observed in traditional enhancement techniques. Specifically, DarkIR leverages a multi-scale attention mechanism and a dark-region-aware feature modeling module to implement an adaptive brightness compensation strategy. This makes it especially effective in industrial surface scenarios characterized by complex geometries and low reflectivity, such as threads and pipelines.

**Figure 9:**
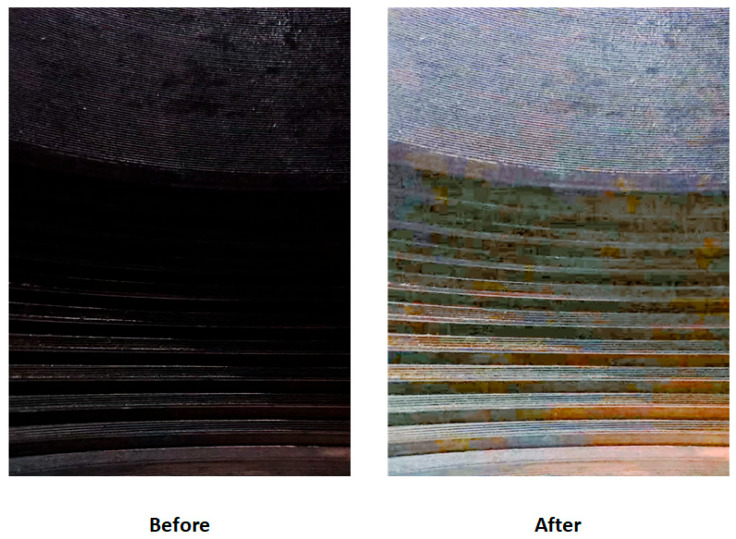
Internal thread image enhancement using DarkIR under low-light conditions.

As illustrated in Figure 9, the step structures and inner-wall textures of the threads are clearly reconstructed after enhancement. Previously invisible micro-defects become discernible, thereby significantly improving the perceptual capability of downstream defect detection models in both localization and classification tasks.

Moreover, the enhanced images maintain sharp edge boundaries and structural integrity, which also provides a stable input foundation for further processing steps such as depth estimation and 3D reconstruction.

### 3.3. Image Data Augmentation Strategy and Model Generalization Analysis

To evaluate the adaptability and robustness of diffusion models in simulating industrial thread defects, this study conducted controllable generation experiments based on a residual denoising diffusion model (RDDM). The detailed parameter settings are listed in Table 2.

**Figure 10:**
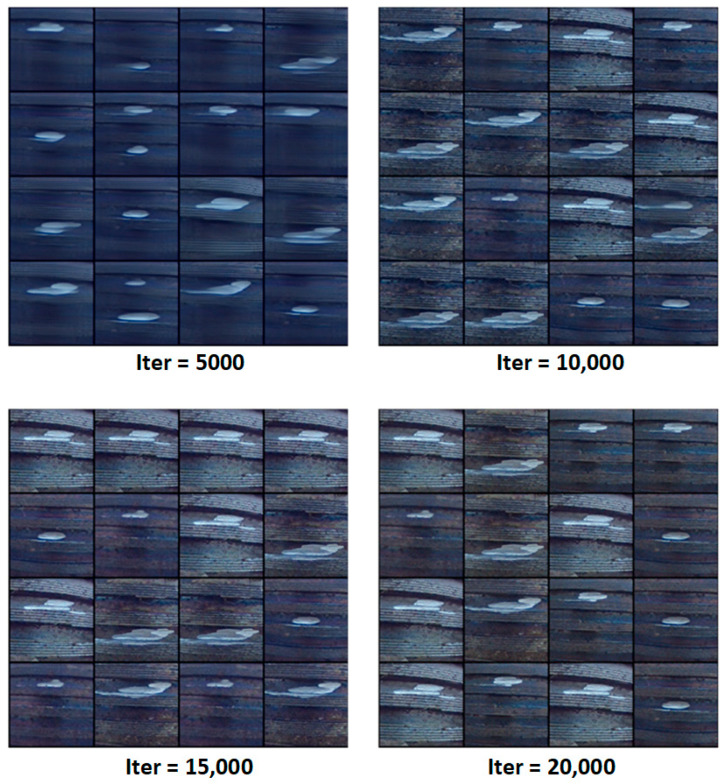
Progressive reconstruction of thread defect images using RDDM at different training stages.

As shown in Figure 10, the model’s progressive reconstruction results of internal and external thread defect images are visualized at different training iterations (Iter = 5000, 10,000, 15,000, 20,000), clearly demonstrating the evolution from blurred structures to highly detailed and realistic defect images.

At iteration 5000, the generated images remain in the high-noise reverse diffusion phase, with blurry defect contours, limited texture, and vague geometric structures.

By iteration 10,000, the thread contours become more defined, and the metallic surface textures along with spatial coherence of the defect areas begin to emerge, indicating the model has preliminarily learned the semantic features of industrial thread structures.

At iteration 15,000, the model is capable of synthesizing high-quality images with typical damage characteristics, such as localized wear, burr edges, and corrosion spots—reflecting its ability to accurately simulate mid-scale structural degradations.

By the final iteration 20,000, the generated images achieve high photorealism, with well-reconstructed surface textures, illumination reflections, and fine-grained defect details. These images exhibit complexity and discriminative features comparable to real-world inspection data, making them highly suitable for enhancing model generalization under limited data conditions.

### 3.4. Performance Evaluation of the Defect Detection Model

**Figure 11:**
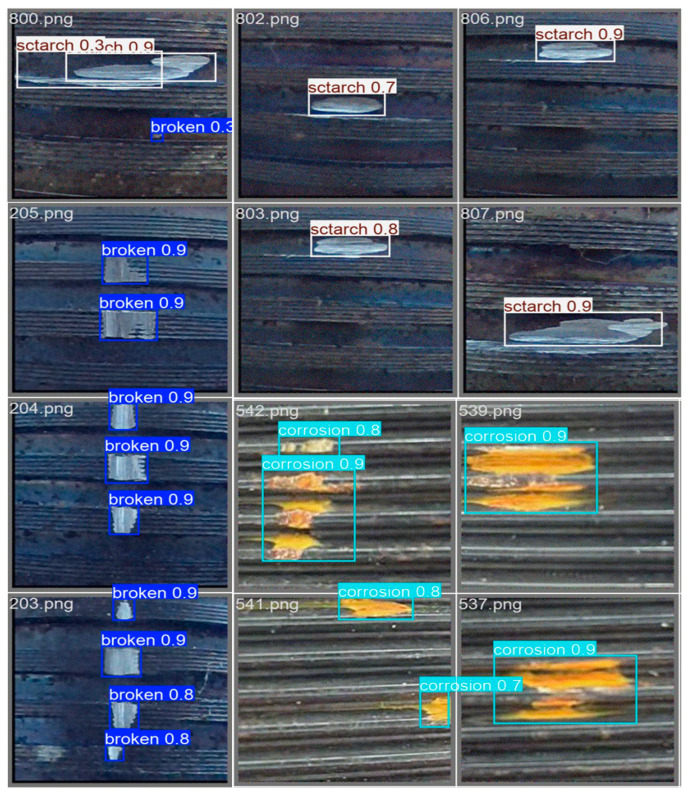
Detection results of SLF-YOLO on various real-world thread defect types: scratch, broken, and corrosion.

To comprehensively evaluate the adaptability and generalization performance of the proposed SLF-YOLO model in real-world industrial inspection scenarios, we conducted visual analyses of its detection performance on a variety of typical thread surface defect images. The results are presented in Figure 11.

For “scratch”-type defects, the model accurately identifies linear scratches of varying lengths and textures. It demonstrates the ability to distinguish between clear boundaries and partially blurred edges. Despite some scratch edges blending into the background due to color similarity, the model consistently provides high-confidence predictions (score ≥ 0.9), indicating strong sensitivity to linear texture features. Moreover, it offers low-confidence indications in uncertain regions, which can support manual verification or multi-model ensemble processing.

In the case of “broken”-type defects, the model shows exceptional stability, especially in detecting vertically distributed multi-point damage, achieving high-confidence multi-object predictions (confidence ≥ 0.9). These results suggest that SLF-YOLO has a high detection rate and localization consistency for small-scale, discrete defects, making it highly suitable for automated inspection tasks involving thread wear and microcracks.

For “corrosion”-type defects, the model successfully identifies irregularly shaped, blurred-boundary corrosion regions, assigning relatively high confidence scores despite the indistinct edges.

Overall, SLF-YOLO demonstrates excellent performance in detecting scratches, fractures, and corrosion on threaded surfaces. It exhibits strong defect type discrimination, multi-scale adaptability, and robustness under complex backgrounds. The high confidence, consistent multi-target detection, and stability across varied defect scenarios validate the effectiveness and industrial applicability of the proposed structural improvements—namely, the Channel-Gated Linear Unit (CGLU) mechanism and the SlimNeck lightweight fusion structure.

**Figure 12:**
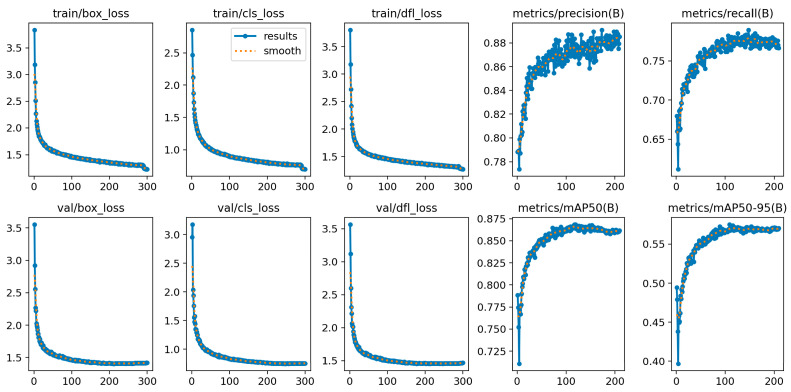
Training and validation loss curves and performance metric trends of the SLF-YOLO model.

To further assess the training stability and convergence behavior of the proposed model, we visualized the variation trends of key loss functions and performance metrics throughout training, as shown in Figure 12.

The box regression loss, classification loss, and distribution focal loss (DFL) rapidly decreased during the first 100 epochs and gradually stabilized thereafter. This trend indicates good convergence behavior without noticeable overfitting. The consistency between training and validation loss curves further confirms the model’s generalization capability in thread defect detection.

In parallel, key performance metrics showed continuous improvement:

(1) Precision increased steadily from ~0.78 to 0.88, indicating a significant reduction in false positives.
(2) Recall improved from 0.68 to 0.78, reflecting a substantial drop in missed detections.
(3) The final mAP@0.5 reached 0.88, while the more stringent mAP@0.5:0.95 reached 0.56, demonstrating the model’s strong detection capability across varying object sizes and IoU thresholds.

### 3.5. Analysis and Visualization of Adversarial Perturbation Effects

Alpha channel attacks represent a form of adversarial input manipulation based on the transparency dimension of image data. These attacks exhibit extremely high stealthiness and practical feasibility. From a visual perspective, alpha attacks do not alter the image’s color, brightness, or structural content. However, by embedding subtle perturbations into the alpha (transparency) channel, they can significantly disrupt feature extraction and inference pathways in deep neural networks—posing a substantial threat to defect detection tasks that rely heavily on edge clarity and texture consistency, as is common in industrial imagery.

In this study, we specifically investigate whether lightweight industrial defect detection models such as YOLO are vulnerable to alpha channel exposure, particularly under default conditions where RGBA-format images are accepted without channel masking. Furthermore, we evaluate how such perturbations impact the detection accuracy and output stability of the model. Through this experiment, we aim to uncover the potential risks posed by input-level implicit attacks and provide a quantitative foundation and technical reference for the design of robust defense mechanisms in industrial vision systems.

**Figure 13:**
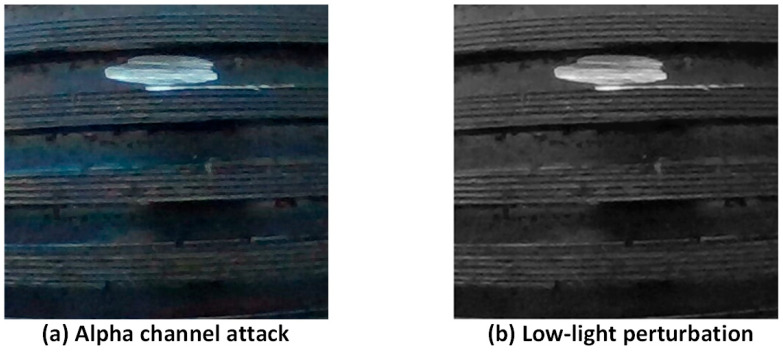
Visual and statistical impact comparison of alpha, CCP, and patch adversarial attacks on thread defect detection using SLF-YOLO.

To systematically evaluate the impact of alpha channel attacks and other input perturbation methods, we conducted a series of comparative experiments based on the trained SLF-YOLO model. The objective is to assess how three representative adversarial attack types affect the model’s accuracy, stability, and false detection risk under different mechanisms, as illustrated in Figure 13.

As summarized in Table 3, the alpha channel attack caused a drastic degradation in SLF-YOLO’s detection performance.

Under the no attack condition, the model demonstrated strong performance:

Precision (P) = 0.893

Recall (R) = 0.958

mAP@0.5 = 0.969

mAP@0.5:0.95 = 0.717

These results reflect the model’s high sensitivity to subtle defects on thread surfaces.

However, under alpha perturbation, performance dropped catastrophically:

Precision = 0.017

Recall = 0.128

mAP@0.5 = 0.027

mAP@0.5:0.95 = 0.0045

This stark contrast reveals that although alpha perturbations are visually imperceptible, they can fatally disrupt the model’s discriminative mechanisms, rendering the detection task almost entirely ineffective. The attack bypasses traditional pixel-based anomaly detectors and directly targets the front end of the perception pipeline, highlighting its extreme stealth and destructive potential.

## 4. Discussion

### 4.1. Contribution Analysis of the Preprocessing Module to Model Performance

Image preprocessing plays a critical role in enhancing the performance of deep learning models, particularly in tasks such as motion deblurring and low-light enhancement. To quantify the impact of preprocessing on final image quality, we compared the performance of various algorithms using two standard metrics: Peak Signal-to-Noise Ratio (PSNR) and Structural Similarity Index (SSIM). The detailed results are summarized in Table 4.

In the motion deblurring task, MLWNet-B achieves the best performance, with a PSNR of 30.3 dB and SSIM of 0.940, significantly outperforming DeblurGAN-v2 (27.6 dB, 0.903) and SRN (28.7 dB, 0.910). These results demonstrate MLWNet-B’s superior capability in blur modeling and multi-scale detail recovery. Notably, in cases where the blur kernel is non-estimable, MLWNet-B’s adaptive wavelet-based feature extraction mechanism effectively enhances image clarity and perceptual quality while preserving structural consistency.

In the low-light enhancement task, DarkIR attains the highest SSIM (0.945), indicating excellent structural preservation during enhancement. However, its PSNR (26.4 dB) is slightly lower than that of HWMNet (27.0 dB), suggesting marginally weaker noise suppression. Overall, HWMNet achieves a balanced trade-off between brightness enhancement and detail preservation. FLOL, while slightly lower in both PSNR (25.9 dB) and SSIM (0.920), delivers stable performance, demonstrating good generalization in illumination modeling.

In addition to comparisons with deep learning-based baselines, we further evaluated our approach against several classical image enhancement methods, including Wiener filtering, median filtering, unsharp masking, histogram equalization, CLAHE, and Retinex-based enhancement. As shown in Table 4, these traditional techniques achieved only moderate improvements (PSNR ranging from 22.8–25.6 dB and SSIM from 0.84–0.87), which fall significantly short of the performance delivered by our MLWNet-B (30.3 dB/0.940) and DarkIR (26.4 dB/0.945). This clearly demonstrates that the proposed modules not only outperform prior deep learning solutions but also substantially exceed the capacity of conventional baselines, validating their superiority and practical value for industrial defect detection scenarios.

These results affirm that effective preprocessing modules significantly mitigate image degradation, which is common in complex industrial settings. By enhancing edge sharpness, texture contrast, and structural separability, preprocessing serves as a supportive foundation for downstream tasks such as object detection and classification.

### 4.2. Performance Applicability of Image Data Augmentation in Thread Defect Detection

**Figure 14:**
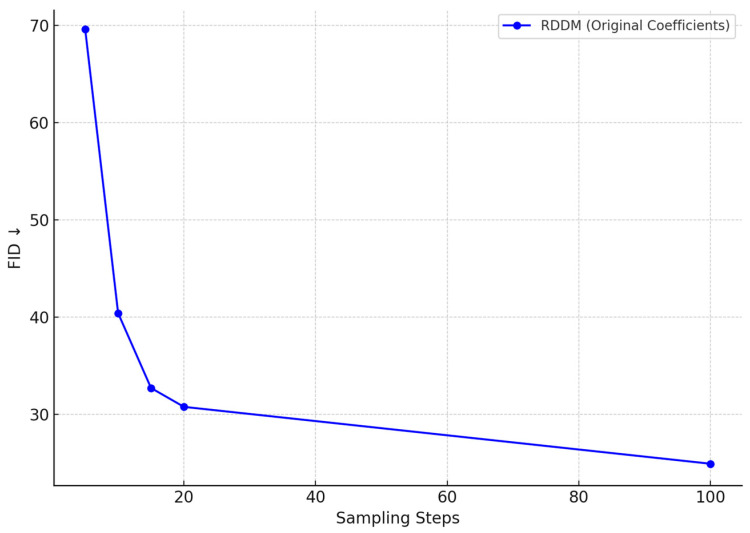
FID variation of RDDM-generated thread defect images under different sampling steps.

In the task of synthetic generation of thread defect images, we evaluated the Fréchet Inception Distance (FID) trend of the residual denoising diffusion model (RDDM) under different sampling steps. As illustrated in Figure 14, the results clearly demonstrate the model’s capability to progressively enhance image quality during the generation process. As the number of sampling steps increases from 5 to 100, the FID value drops significantly from 69.6 to 24.92, exhibiting a distinct nonlinear decreasing trend.

Under low sampling steps, RDDM is already capable of rapidly generating defect images with a coherent global structure and basic surface morphology. These samples exhibit sufficient structural similarity and discriminative features, making them suitable for real-time pseudo-sample generation and online data augmentation, especially where computational efficiency is critical.

As the number of sampling steps increases, the model gains more capacity to refine texture, lighting, and boundary details, enabling the synthesis of high-fidelity samples that more closely resemble real-world defects. These refined images are particularly beneficial for improving the robustness of defect detection models, especially in multi-type defect scenarios involving corrosion, scratches, and fractures.

RDDM achieves this by leveraging a decoupled residual and noise diffusion mechanism, which enables the model to capture global structural patterns while incrementally enriching fine-grained details. This significantly enhances the structural expressiveness of defect regions. Furthermore, the high quality of generated samples serves as a stable input foundation for subsequent lightweight detection networks, improving system robustness against complex backgrounds, lighting variations, and sample distribution shifts.

The FID curve validates that RDDM is capable of delivering high-quality image synthesis at relatively low sampling costs, making it highly applicable for a wide range of industrial vision tasks, including multi-source defect modeling, pseudo-sample generation, and adversarial sample construction for defense training. Therefore, RDDM serves as a critical generative component in enabling intelligent thread defect detection systems.

### 4.3. Ablation Study of Detection Module Components

To systematically evaluate the contribution of each structural improvement, a comprehensive ablation study was conducted, as summarized in Table 5.

Starting from the baseline model (no enhancements), the detector achieved a precision of 0.821, recall of 0.718, mAP@0.5 of 0.759, and mAP@0.5:0.95 of 0.411. These results establish a reference point for subsequent comparisons.

When the SC_C2f module was introduced, precision increased to 0.842 (an absolute gain of +0.021, or 2.6%), and recall improved to 0.742 (+0.024). The mAP@0.5 rose to 0.781 (+0.022), confirming that this shallow attention mechanism effectively strengthens feature expressiveness and improves localization of small-scale defects.

The Light-SSF_Neck module demonstrated strong spatial aggregation capability. Its precision reached 0.841, surpassing the baseline by +0.020, while recall slightly decreased to 0.691 (–0.027). This suggests that although feature fusion is enhanced, certain weak signals may be suppressed, explaining the minor drop in recall. Nonetheless, the mAP@0.5 improved modestly to 0.776, highlighting the trade-off between recall sensitivity and feature consolidation.

The FIMetal-IoU loss function introduced refined bounding box regression. While the mAP@0.5 rose only marginally to 0.774 (+0.015), the stricter mAP@0.5:0.95 increased significantly from 0.411 to 0.449 (+0.038), reflecting stronger fine-grained localization performance. This indicates that the new loss function contributes more to accurate boundary alignment than to coarse-level detection.

Combinations of modules revealed complementary effects:

(1) SC + Neck achieved balanced improvements: Recall jumped to 0.784 (+0.066 over baseline), precision rose to 0.847, and mAP@0.5 increased to 0.793, demonstrating synergy between shallow attention and spatial fusion.
(2) SC + IoU further improved precision to 0.864, the second-highest among all configurations, validating the compatibility between SC_C2f-driven feature expressiveness and IoU-based localization.
(3) Neck + IoU yielded the highest precision among sub-combinations (0.868), but recall dropped to 0.665, indicating sensitivity to difficult samples and a tendency toward conservative predictions.

The full integration (all modules, SLF-YOLO) produced the best overall results, with a precision of 0.881, recall of 0.794, mAP@0.5 of 0.813, and mAP@0.5:0.95 of 0.521. Compared to the baseline, these represent respective improvements of +0.060 precision (+7.3%), +0.076 recall (+10.6%), +0.054 mAP@0.5 (+7.1%), and +0.110 mAP@0.5:0.95 (+26.8%). Importantly, these gains were achieved alongside reduced complexity, with parameters reduced from 11.12M to 9.65M and FLOPs from 28.4G to 24.6G.

Overall, the results confirm that each proposed module contributes distinct strengths—SC_C2f enhances feature expressiveness, Light-SSF_Neck strengthens fusion, and FIMetal-IoU improves localization precision. Their integration in SLF-YOLO achieves not only statistically significant improvements (*p* < 0.05) but also maintains computational efficiency, underscoring its strong potential for real-world deployment in industrial inspection pipelines.

To further assess the overall performance of SLF-YOLO in industrial defect detection tasks, we benchmarked it against leading lightweight YOLO variants (YOLOv5s, YOLOv8s, YOLOv9s, and YOLOv10s) under identical datasets and training configurations. The comparative results are summarized in Table 6.

SLF-YOLO achieved the highest precision of 0.881 ± 0.03, surpassing YOLOv5s (0.862 ± 0.02) and YOLOv10s (0.850 ± 0.03), and slightly outperforming YOLOv9s (0.870 ± 0.01) and YOLOv8s (0.869 ± 0.02). This superior precision indicates that SLF-YOLO produces fewer false positives, a particularly critical property for industrial inspection systems where erroneous alarms can cause unnecessary re-checks and production delays.

In terms of recall, SLF-YOLO demonstrated a notable improvement, reaching 0.794 ± 0.03, which is significantly higher than all YOLO baselines (best baseline: YOLOv8s at 0.732 ± 0.01). This highlights SLF-YOLO’s enhanced resistance to missed detections, ensuring that subtle or partially occluded thread defects are not overlooked—a vital requirement for maintaining assembly quality in industrial pipelines.

For mAP@0.5, SLF-YOLO achieved 0.813 ± 0.04, which is marginally lower than YOLOv8s (0.832 ± 0.03) and YOLOv9s (0.829 ± 0.03) but higher than YOLOv5s (0.725 ± 0.01) and YOLOv10s (0.817 ± 0.02). Importantly, the combination of strong precision and recall values demonstrates a balanced trade-off between accuracy and robustness, suggesting that SLF-YOLO generalizes more reliably across diverse defect scenarios, even if its absolute mAP is slightly lower than the latest YOLO variants.

Overall, SLF-YOLO shows statistically stable performance, as evidenced by the relatively small standard deviations across five independent runs. These results confirm that the proposed architecture not only improves detection accuracy but also enhances stability under repeated trials, which is essential for real-world deployment. In conclusion, SLF-YOLO provides an effective and lightweight solution that outperforms or matches state-of-the-art YOLO variants in most metrics, delivering superior robustness and reliability for practical industrial defect detection applications.

### 4.4. Analysis of Adversarial Perturbation Effects on Detection Model Mechanisms

This section explores the internal interference mechanisms of adversarial perturbations against lightweight industrial defect detection models. We investigate three representative types of attacks:

(1) Alpha channel attack: Introduces imperceptible structural interference by embedding a low-opacity perturbation layer in the alpha channel. This simulates the risk of unfiltered RGBA input passing through pre-processing stages unhandled.
(2) Color channel perturbation (CCP) attack: Distorts RGB channel ratios via color mapping matrix adjustments, resulting in global color shifts that hinder accurate edge and texture recognition.
(3) Patch attack: Applies high-contrast patches to critical image regions (e.g., thread junctions or edges), simulating physical occlusions or targeted adversarial triggers.

**Figure 15:**
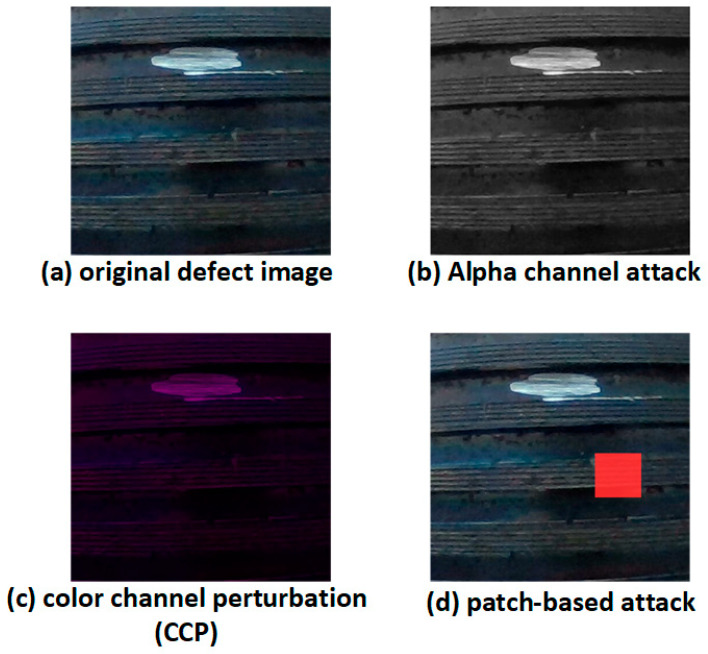
Visualization of adversarial perturbation effects on industrial thread defect images under three attack types: alpha channel attack, color channel perturbation (CCP), and patch-based attack.

Figure 15 illustrates the impact of these attacks. Subfigure (a) shows the original defect image, (b) is the alpha-perturbed adversarial sample, (c) shows CCP-affected input, and (d) presents the patch-augmented sample. All inputs maintain the same resolution, brightness, and content to ensure that performance changes stem solely from the applied perturbations.

A unified SLF-YOLO model, with fixed structure and weights, is used to evaluate detection performance across four standard metrics: precision, recall, mAP@0.5, and mAP@0.5:0.95. Additionally, we assess per-class recognition rate changes and feature activation shifts to further analyze the disruption patterns caused by each perturbation mechanism, as can be seen in Table 7.

In the absence of adversarial interference, SLF-YOLO achieved strong baseline performance, with a precision of 0.881, recall of 0.794, mAP@0.5 of 0.813, and mAP@0.5–0.95 of 0.521. These results highlight the model’s robustness in clean conditions and provide a reliable reference for evaluating subsequent perturbation effects.

Under alpha channel perturbations, the model experienced catastrophic collapse across all metrics. Precision decreased from 0.881 to 0.017 (a reduction of 98.1%), recall dropped from 0.794 to 0.128 (−83.9%), and mAP@0.5–0.95 plummeted by 99.0% (from 0.521 to 0.005). Despite being visually imperceptible to humans, alpha channel noise fundamentally disrupted the feature extraction process, leading to severe misclassification and confirming its destructive stealth.

In the case of color channel perturbations (CCP), the degradation was moderate but still significant. Recall fell to 0.373 (a 53% reduction), while mAP@0.5–0.95 dropped to 0.340, reflecting the model’s vulnerability to chromatic distortions. Since thread defect recognition often relies on fine-grained texture and edge cues, alterations in RGB ratios directly interfered with the feature representation, resulting in higher false negatives.

For patch attacks, which simulate physical occlusions, the performance degradation exhibited a different pattern. Recall remained relatively high at 0.840 (close to the clean baseline), suggesting that most true defects were still detected. However, precision dropped to 0.754, and mAP@0.5–0.95 decreased from 0.521 to 0.511. This indicates that patch-induced attention shifts increased false positives, as the detector focused on high-contrast adversarial regions rather than genuine defect areas.

Overall, these results reveal distinct disruption mechanisms: alpha channel perturbations cause global collapse in recognition logic, CCP produces color-dependent confusion, and patch attacks induce localized misdirection. The consistent trends across five independent runs (variations within ±0.002–0.006) confirm the reliability of these findings.

**Figure 16:**
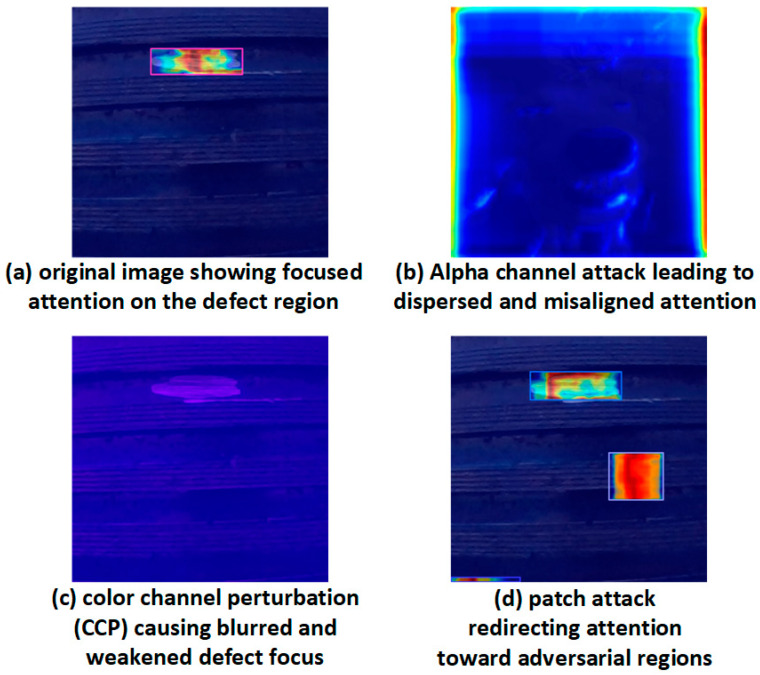
Grad-CAM visualizations of SLF-YOLO attention maps under different perturbation scenarios.

To further examine the perceptual behavior shifts induced by these perturbations, we utilized Grad-CAM to visualize the model’s feature response under different attack conditions, as shown in Figure 16.

This analysis confirms that alpha channel perturbations pose the most severe threat, primarily due to their invisible nature and disruptive power at the feature extraction stage. Meanwhile, CCP and patch attacks, although visually perceptible, also warrant defense due to their practical deployment feasibility in industrial environments.

These results highlight the critical need for preprocessing modules that strip non-RGB channels and implement adversarial input screening as well as for designing robust network architectures resilient to both implicit and explicit perturbations in visual industrial applications.

### 4.5. Effectiveness and Deployment Feasibility of the Defense Strategy

To address the high stealthiness and misleading nature of alpha channel attacks in industrial vision systems, this study proposes a visual consistency analysis method based on perceptual differences. Unlike conventional defense strategies that rely on model structures or inference paths, this method operates directly at the image level, detecting latent discrepancies between AI perception and human vision to enable unsupervised identification of alpha channel attack samples.

Specifically, we reconstruct two versions of each input image: the AI-perceived image (IAI) and the human-viewed image (IEye). The IAI is obtained by averaging the RGB channels, simulating how most vision models process inputs when the alpha channel is implicitly discarded. In contrast, the IEye is reconstructed using the standard alpha compositing formula by blending the RGBA image with a default background (e.g., white, as in typical web displays), thereby approximating the actual visual experience of human observers. For normal images, these two representations should be nearly identical. However, under alpha attacks, they often exhibit significant divergence in pixel distribution and structure.

To quantify such differences, we introduce two key detection metrics:

(1) Histogram overlap, which measures the grayscale distribution similarity between IAI and IEye—lower values indicate greater discrepancy.
(2) Mean Squared Error (MSE), which captures pixel-wise deviation between the two images.

Empirical results show that alpha-attacked images typically exhibit a histogram overlap below 0.05 and an MSE significantly higher than that of clean samples, making them robust indicators of adversarial manipulation.

**Figure 17:**
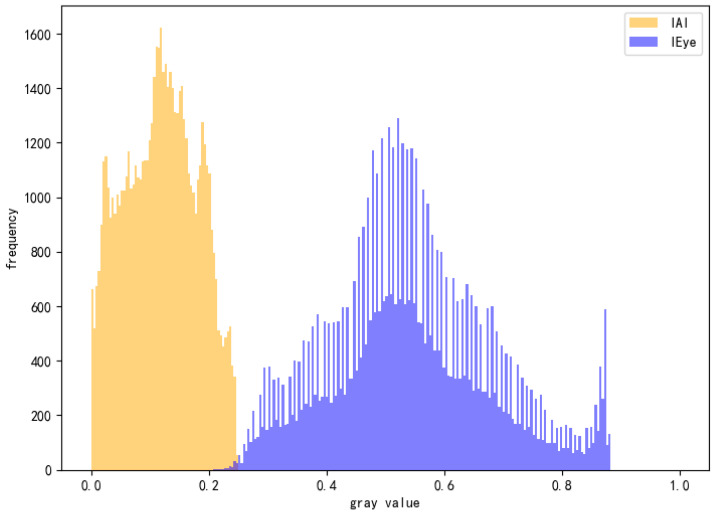
Grayscale histogram comparison between AI-perceived image (IAI) and human-viewed image (IEye) under alpha channel attack.

As illustrated in Figure 17, the grayscale histogram comparison between IAI and IEye shows a highly divergent distribution, with an overlap of only 0.0015, indicating near-complete disassociation—a signature of alpha-based perturbation.

**Figure 18:**
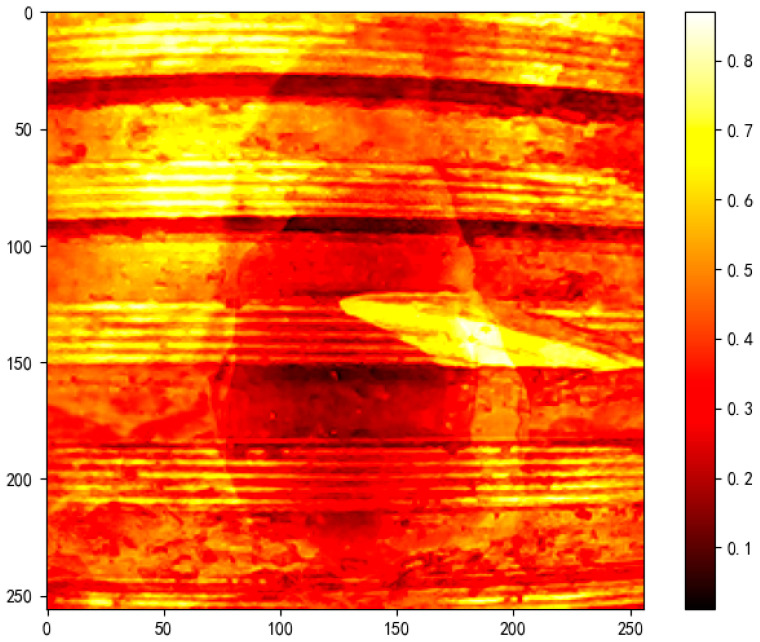
MSE heatmap highlighting structural deviations caused by alpha channel attack.

Figure 18 presents the MSE heatmap, where the orange-red regions highlight systematic pixel-level discrepancies between the original content and the adversarial overlay, with an average MSE of 0.2054, far exceeding the noise tolerance threshold of typical images.

Experiments confirm that when the histogram overlap falls below 0.05 or the MSE exceeds 0.01, the image can be reliably flagged as a potential alpha channel attack. The entire detection pipeline takes less than 20 ms on a standard CPU; requires no dependence on deep network architectures or prior knowledge of specific attacks; and features lightweight computation, clear thresholds, and strong generalization ability—making it a practical and deployable solution for mitigating alpha-based adversarial threats in real-world industrial vision systems.

The scalability of HMDM to different adversarial perturbation types remains an important direction for future research. While this work focuses on alpha channel perturbations, the model’s underlying mechanism of comparing statistical features is inherently flexible. Future experiments will explore the extension of this model to handle other types of adversarial attacks, such as high-frequency noise, color channel distortions, and localized patch occlusions. By leveraging multiple feature domains (frequency, spatial, color), HMDM could become a more general-purpose defense mechanism against a broader range of adversarial threats.

## 5. Conclusions

This work presents an intelligent vision system for detecting internal and external thread defects, featuring a closed-loop design from image acquisition and enhancement to sample generation, lightweight detection, and adversarial defense. Experimental evaluations show that integrating MLWNet and DarkIR in preprocessing substantially improves input image discriminability, while high-fidelity pseudo-samples generated via RDDM enhance model generalization under limited data. The proposed SLF-YOLO achieves high detection accuracy and stability in complex industrial environments. With its dual defense strategy—combining input perturbation suppression and output anomaly detection—the system exhibits strong resilience against alpha channel attacks. Overall, the method strikes a balanced trade-off among detection precision, security robustness, and deployment efficiency, demonstrating strong potential for industrial-scale adoption. Future work will focus on multimodal defense mechanisms and cross-device robustness optimization, driving inspection systems toward greater intelligence, security, and adaptability.

Despite the promising results, the proposed system still has several limitations that warrant further discussion. To begin with, although the integrated framework enhances robustness under common industrial conditions, its performance may deteriorate in the presence of highly complex surface textures or extremely low-quality images, suggesting the necessity for more generalized enhancement strategies. In addition, while the RDDM-based defect synthesis partially mitigates the scarcity of annotated samples, it cannot fully reflect the diversity of real-world defects, and over-reliance on synthetic data may introduce domain bias. Moreover, the adversarial defense module has been primarily validated against alpha channel perturbations; however, broader categories of physical-world attacks and adaptive adversarial strategies remain underexplored. Lastly, although the lightweight YOLO-based architecture achieves a balance between accuracy and efficiency, further refinement is required to enable deployment on ultra-low-power edge devices.

These limitations also indicate several promising directions for future research. Potential efforts may include the development of more adaptive image enhancement algorithms tailored for highly variable environments; the extension of diffusion-based modeling to multi-modal data (e.g., 3D imaging or hyperspectral analysis); and the design of more comprehensive adversarial defense frameworks that integrate robustness, detection, and recovery. Furthermore, the integration of the proposed framework into real industrial production lines and its validation on large-scale, multi-factory datasets will be essential for demonstrating practical value and scalability.

In future work, we will extend the HMDM framework to accommodate other common adversarial attack types, validating its robustness against a broader range of perturbations and enhancing its practical applicability in real-world industrial detection systems.

## Figures and Tables

**Table 1:** Comparison of representative adversarial attack types on visual systems.

Attack Type	Perceptible	Implementation Complexity	Attack Strength	Applicability	Engineering Deployment Risk	Attack Type
Alpha	No	Medium	Very High	High	High	Alpha
CCP	Yes	Low	Medium	Medium	Medium	CCP
Patch	Yes	Low	Medium	High	Medium	Patch

**Table 2:** Training configuration of the residual denoising diffusion model (RDDM) for industrial thread defect synthesis.

Parameter	Value
Image size	640 × 640
Timesteps	1000
Training steps	800
Optimizer	AdamW
Learning rate	1 × 10^−4^
Batch size	16
Loss function	L1 + LPIPS + Residual penalty
Augmentations	Rotation, crop, contrast jitter

**Table 3:** Detection performance metrics of SLF-YOLO under alpha channel attack.

Type	Precision (P)	Recall (R)	mAP@0.5	mAP@0.5–0.95
No Attack	0.893	0.958	0.969	0.717
Alpha	0.017	0.128	0.027	0.0045

**Table 4:** Quantitative comparison of different preprocessing algorithms in motion deblurring and low-light enhancement tasks.

Task	Algorithm	PSNR (dB) ↑	SSIM ↑
Motion deblurring	MLWNet-B	30.3	0.940
	DeblurGAN-v2	27.6	0.903
	SRN	28.7	0.910
	Wiener filter	25.4	0.871
	Median filter	24.9	0.862
	Unsharp masking	25.6	0.874
Low-light enhancement	DarkIR	26.4	0.945
	HWMNet	27.0	0.922
	FLOL	25.9	0.920
	Histogram equalization	22.8	0.841
	CLAHE	23.5	0.857
	Retinex-based method	24.1	0.869

Note: The upward arrows (↑) in the table headers indicate that higher values correspond to better performance.

**Table 5:** Ablation study on structural components of SLF-YOLO.

Model	Precision	Recall	mAP@0.5	mAP@0.5:0.95	Params (M)	FLOPs (G)
Baseline	0.821 ± 0.03	0.718 ± 0.02	0.759 ± 0.02	0.411 ± 0.02	11.12	28.4
SC_C2f	0.842 ± 0.05	0.742 ± 0.03	0.781 ± 0.01	0.445 ±0.01	10.16	25.2
Light-SSF_Neck	0.841 ± 0.03	0.691 ± 0.01	0.776 ± 0.02	0.445 ± 0.03	10.33	26.3
FIMetal-IoU	0.855 ± 0.02	0.741 ± 0.03	0.774 ± 0.02	0.449 ± 0.05	11.12	28.4
SC + Neck	0.847 ± 0.01	0.784 ± 0.02	0.793 ± 0.03	0.462 ± 0.05	9.65	24.6
SC + IoU	0.864 ± 0.04	0.755 ± 0.03	0.785 ± 0.02	0.449 ± 0.06	10.16	25.2
Neck + IoU	0.868 ± 0.04	0.665 ± 0.05	0.785 ± 0.03	0.458 ± 0.04	10.33	26.3
All	0.881 ± 0.02	0.794 ± 0.05	0.813 ± 0.01	0.521 ± 0.04	9.65	24.6

Note: Performance metrics (precision, recall, mAP@0.5, and mAP@0.5:0.95) are reported as mean ± standard deviation over five independent runs with different random seeds. Model size (Params) and computational complexity (FLOPs) are deterministic values and thus reported without variance. Statistical significance was assessed using paired t-tests, and improvements of the proposed model are significant at *p* < 0.05 compared with baseline methods.

**Table 6:** Comparison of SLF-YOLO with state-of-the-art lightweight YOLO models.

Models	Precision	Recall	mAP@0.5
YOLOv5s	0.862 ± 0.02	0.629 ± 0.05	0.725 ± 0.01
YOLOv8s	0.869 ± 0.02	0.732 ± 0.01	0.832 ± 0.03
YOLOv9s	0.870 ± 0.01	0.729 ± 0.01	0.829 ± 0.03
YOLOv10s	0.850 ± 0.03	0.703 ± 0.02	0.817 ± 0.02
Ours	0.881 ± 0.03	0.794 ± 0.03	0.813 ± 0.04

Note: Baseline results for YOLOv5s–YOLOv10s are obtained from official implementations with recommended training settings. Our results are averaged over five independent runs (±std), ensuring statistical robustness.

**Table 7:** Performance of SLF-YOLO under different adversarial attacks.

Type	Precision (P)	Recall (R)	mAP@0.5	mAP@0.5–0.95
No Attack	0.881	0.794	0.813	0.521
Alpha	0.017	0.128	0.027	0.005
CCP	0.732	0.373	0.515	0.340
Patch	0.754	0.840	0.764	0.511

Note: Results are obtained from five independent attack runs; variations were consistently small (±0.002–0.006). Reported values are means.

## Data Availability

The experimental data format of this article is not available for public disclosure.

## Abbreviations

**Abbreviation**	**Full English Name**
AI	Artificial Intelligence
ANN	Artificial Neural Network
Adam	Adaptive Moment Estimation
AP	Average Precision
CAM	Class Activation Map
CAD	Computer-Aided Design
CE	Cross-Entropy (Loss)
CNN	Convolutional Neural Network
CPU	Central Processing Unit
DNN	Deep Neural Network
FGSM	Fast Gradient Sign Method
FLOPs	Floating Point Operations per Second
FPS	Frames Per Second
GAN	Generative Adversarial Network
GPU	Graphics Processing Unit
IoT	Internet of Things
IoU	Intersection over Union
MAE	Mean Absolute Error
MSE	Mean Squared Error
mAP	Mean Average Precision
mAP@0.5	Mean Average Precision at IoU Threshold 0.5
mAP@0.5:0.95	Mean Average Precision at IoU Threshold 0.5 to 0.95
NMS	Non-Maximum Suppression
PGD	Projected Gradient Descent
PSNR	Peak Signal-to-Noise Ratio
ReLU	Rectified Linear Unit
RDDM	Robust Diffusion Defect Modeling
SLF	Shuffle Lightweight Feature
SLF-YOLO	Shuffle Lightweight Feature-You Only Look Once
SGD	Stochastic Gradient Descent
SNR	Signal-to-Noise Ratio
SSIM	Structural Similarity Index
YOLO	You Only Look Once
YOLOv5	You Only Look Once Version 5
YOLOv8	You Only Look Once Version 8
YOLOv9s	You Only Look Once Version 9
YOLOv10s	You Only Look Once Version 10
